# Serum Uric Acid/Serum Creatinine Ratio and Cardiovascular Mortality in Diabetic Individuals—The Uric Acid Right for Heart Health (URRAH) Project

**DOI:** 10.3390/metabo14030164

**Published:** 2024-03-14

**Authors:** Lanfranco D’Elia, Maria Masulli, Pietro Cirillo, Agostino Virdis, Edoardo Casiglia, Valerie Tikhonoff, Fabio Angeli, Carlo Maria Barbagallo, Michele Bombelli, Federica Cappelli, Rosario Cianci, Michele Ciccarelli, Arrigo F. G. Cicero, Massimo Cirillo, Raffaella Dell’Oro, Giovambattista Desideri, Claudio Ferri, Loreto Gesualdo, Cristina Giannattasio, Guido Grassi, Guido Iaccarino, Luciano Lippa, Francesca Mallamaci, Alessandro Maloberti, Stefano Masi, Alberto Mazza, Alessandro Mengozzi, Maria Lorenza Muiesan, Pietro Nazzaro, Paolo Palatini, Gianfranco Parati, Roberto Pontremoli, Fosca Quarti-Trevano, Marcello Rattazzi, Gianpaolo Reboldi, Giulia Rivasi, Elisa Russo, Massimo Salvetti, Giuliano Tocci, Andrea Ungar, Paolo Verdecchia, Francesca Viazzi, Massimo Volpe, Claudio Borghi, Ferruccio Galletti

**Affiliations:** 1 Department of Clinical Medicine and Surgery, “Federico II” University of Naples, 80131 Naples, Italy; lanfranco.delia@unina.it (L.D.); maria.masulli@unina.it (M.M.); guiaccar@unina.it (G.I.); 2 Nephrology, Dialysis and Transplantation Unit, Department of Emergency and Organ Transplantation, “Aldo Moro” University of Bari, 70122 Bari, Italy; pietro.cirillo@policlinico.ba.it (P.C.); loreto.gesualdo@uniba.it (L.G.); 3 Department of Clinical and Experimental Medicine, University of Pisa, 56126 Pisa, Italy; agostino.virdis@unipi.it (A.V.); federica.cappelli@med.unipi.it (F.C.); stefano.masi@unipi.it (S.M.); alessandro.mengozzi@unipi.it (A.M.); 4 Studium Patavinum, Department of Medicine, University of Padua, 35100 Padua, Italy; edoardo.casiglia@unipd.it (E.C.); palatini@unipd.it (P.P.); 5 Department of Medicine, University of Padua, 35100 Padua, Italy; valerie.tikhonoff@unipd.it; 6 Department of Medicine and Technological Innovation (DiMIT), University of Insubria, 21100 Varese, Italy; fabio.angeli@uninsubria.it; 7 Department of Medicine and Cardiopulmonary Rehabilitation, Maugeri Care and Research Institutes, IRCCS, 21049 Tradate, Italy; 8 Biomedical Department of Internal Medicine and Specialistics, University of Palermo, 90100 Palermo, Italy; carlo.barbagallo@unipa.it; 9 Internal Medicine, Pio XI Hospital of Desio, ASST Brianza, University of Milano-Bicocca, 20871 Vimercate, Italy; michele.bombelli@unimib.it; 10 Department of Translational and Precision Medicine, Sapienza University of Rome, 00185 Rome, Italy; rosario.cianci@uniroma1.it; 11 Department of Medicine and Surgery, University of Salerno, 84081 Salerno, Italy; mciccarelli@unisa.it; 12 Hypertension and Cardiovascular Disease Research Center, Medical and Surgical Sciences Department, Alma Mater Studiorum University of Bologna, 40126 Bologna, Italy; arrigo.cicero@unibo.it (A.F.G.C.); claudio.borghi@unibo.it (C.B.); 13 Cardiovascular Medicine Unit, Heart-Chest-Vascular Department, IRCCS Azienda Ospedaliero-Universitaria di Bologna, 40126 Bologna, Italy; 14 Department of Public Health, “Federico II” University of Naples, 80131 Naples, Italy; mcirillo@unisa.it; 15 Clinica Medica, Department of Medicine and Surgery, University of Milano-Bicocca, 20900 Monza, Italy; raffaella.delloro@unimib.it (R.D.); guido.grassi@unimib.it (G.G.); fosca.quarti@unimib.it (F.Q.-T.); 16 Department of Clinical, Internal Medicine, Anesthesiologic and Cardiovascular Sciences, Sapienza University of Rome, 00161 Rome, Italy; giovambattista.desideri@cc.univaq.it; 17 Department of Life, Health and Environmental Sciences, University of L’Aquila, 67100 L’Aquila, Italy; claudio.ferri@univaq.it; 18 Cardiology IV, “A.De Gasperi’s” Department, Niguarda Ca’ Granda Hospital, 20162 Milan, Italy; cristina.giannattasio@unimib.it (C.G.); alessandro.maloberti@ospedaleniguarda.it (A.M.); 19 School of Medicine and Surgery, Milano-Bicocca University, 20126 Milan, Italy; 20 Italian Society of General Medicine (SIMG), 67051 Avezzano, Italy; lippa.luciano@simg.it; 21 Department of Nephrology, Dialysis and Transplantation GOM “Bianchi-Melacrino-Morelli”, 89133 Reggio Calabria, Italy; francesca.mallamaci@ospedalerc.it; 22 CNR-IFC, Institute of Clinical Physiology, Research Unit of Clinical Epidemiology and Physiopathology of Renal Diseases and Hypertension, 89133 Reggio Calabria, Italy; 23 Department of Internal Medicine, Santa Maria Della Misericordia General Hospital, AULSS 5 Polesana, 45100 Rovigo, Italy; alberto.mazza@aulss5.veneto.it; 24 Center for Translational and Experimental Cardiology (CTEC), Department of Cardiology, University Hospital Zurich, University of Zurich, 8952 Schlieren, Switzerland; 25 Health Science Interdisciplinary Center, Scuola Superiore Sant’Anna, 56127 Pisa, Italy; 26 Department of Clinical and Experimental Sciences, University of Brescia, 25121 Brescia, Italy; marialorenza.muiesan@unibs.it (M.L.M.); massimo.salvetti@unibs.it (M.S.); 27 Department of Precision and Regenerative Medicine and Jonic Area (DiMePRe-J), Neurosciences and Sense Organs, University of Bari Medical School, 70122 Bari, Italy; pietro.nazzaro@uniba.it; 28 San Luca Hospital, IRCCS, Istituto Auxologico Italiano, 20149 Milan, Italy; gianfranco.parati@unimib.it; 29 Department of Medicine and Surgery, University of Milano-Bicocca, 20126 Milan, Italy; 30 Dipartimento di Medicina Interna e Specialità Mediche, Università degli Studi di Genova, 16132 Genova, Italy; roberto.pontremoli@unige.it (R.P.); elisa.russo@unige.it (E.R.); francesca.viazzi@unige.it (F.V.); 31 IRCCS Ospedale Policlinico San Martino, 16132 Genova, Italy; 32 Medicina Interna 1°, Ca’ Foncello, Department of Medicine—DIMED, University of Padova Hospital, 31100 Treviso, Italy; marcello.rattazzi@unipd.it; 33 Department of Medicine and Surgery, University of Perugia, 06100 Perugia, Italy; paolo.reboldi@unipg.it; 34 Department of Geriatric and Intensive Care Medicine, Careggi Hospital, University of Florence, 50121 Florence, Italy; giulia.rivasi@unifi.it (G.R.); andrea.ungar@unifi.it (A.U.); 35 Department of Clinical and Molecular Medicine, University of Rome Sapienza, 00189 Rome, Italy; giuliano.tocci@uniroma1.it (G.T.); massimo.volpe@uniroma1.it (M.V.); 36 Hypertension Unit, Division of Cardiology, Sant’Andrea Hospital, 00189 Rome, Italy; 37 Hospital S. Maria della Misericordia, 06100 Perugia, Italy; auci.perugia@tin.it; 38 IRCCS San Raffaele, 00163 Roma, Italy

**Keywords:** uric acid, creatinine, cardiovascular, diabetes

## Abstract

Several studies have detected a direct association between serum uric acid (SUA) and cardiovascular (CV) risk. In consideration that SUA largely depends on kidney function, some studies explored the role of the serum creatinine (sCr)-normalized SUA (SUA/sCr) ratio in different settings. Previously, the URRAH (URic acid Right for heArt Health) Study has identified a cut-off value of this index to predict CV mortality at 5.35 Units. Therefore, given that no SUA/sCr ratio threshold for CV risk has been identified for patients with diabetes, we aimed to assess the relationship between this index and CV mortality and to validate this threshold in the URRAH subpopulation with diabetes; the URRAH participants with diabetes were studied (*n* = 2230). The risk of CV mortality was evaluated by the Kaplan–Meier estimator and Cox multivariate analysis. During a median follow-up of 9.2 years, 380 CV deaths occurred. A non-linear inverse association between baseline SUA/sCr ratio and risk of CV mortality was detected. In the whole sample, SUA/sCr ratio > 5.35 Units was not a significant predictor of CV mortality in diabetic patients. However, after stratification by kidney function, values > 5.35 Units were associated with a significantly higher mortality rate only in normal kidney function, while, in participants with overt kidney dysfunction, values of SUA/sCr ratio > 7.50 Units were associated with higher CV mortality. The SUA/sCr ratio threshold, previously proposed by the URRAH Study Group, is predictive of an increased risk of CV mortality in people with diabetes and preserved kidney function. While, in consideration of the strong association among kidney function, SUA, and CV mortality, a different cut-point was detected for diabetics with impaired kidney function. These data highlight the different predictive roles of SUA (and its interaction with kidney function) in CV risk, pointing out the difference in metabolic- and kidney-dependent SUA levels also in diabetic individuals.

## 1. Introduction

Several studies have detected a direct association between serum uric acid (SUA) and cardiovascular (CV) risk. In particular, the URic acid Right for heArt Health (URRAH) Study, an Italian multicenter cohort study, has unequivocally shown that SUA is an independent risk factor for all-cause and CV mortality in different settings [1,2]. Given that SUA largely depends on kidney function, a number of studies explored the role of the kidney function-normalized SUA (SUA to creatinine ratio—SUA/sCr) [3,4,5,6,7]. This ratio is associated with metabolic diseases (e.g., metabolic syndrome, non-alcoholic fatty liver disease, β-cell dysfunction), chronic obstructive pulmonary disease, and also with increased all-cause mortality [4,6,7,8,9]. Recently, in the URRAH population, a threshold for CV risk was identified for the general population; in particular, a SUA/sCr ratio above 5.35 Units was an independent predictor of CV mortality both in men and women [10]. Therefore, given (i) the strong interaction among diabetes, kidney function, and SUA [11], (ii) few available data on the CV predictive role of SUA/sCr ratio in diabetic patients, (iii) the low cost of the SUA/sCr ratio and its reliability applicable to routine clinical practice of diabetes care, we aimed to assess the relationship between this index and CV mortality and to validate, the SUA/sCr ratio threshold of 5.35 Units (previously proposed for the risk of CV mortality in the general population [10]), in the subgroup of diabetic individuals of the URRAH study population.

## 2. Materials and Methods (See Supplemental Methods)

### 2.1. Study Population

The URRAH database is a multicenter retrospective, observational cohort study, which involves data from several cohorts distributed in almost all the Italian regions (age: 18–95 years). All the details of the URRAH project have been published previously [12]. From an updated URRAH database (a total of 3157 diabetic participants at baseline—12% of the whole population), 2230 diabetic participants were considered for the purpose of the present study after sequential exclusion of participants without a complete database (*n* = 903), and those on hypouricemic therapy at baseline (*n* = 24).

### 2.2. Examination Procedures and Outcomes Assessment

The URRAH study procedures have been extensively described [12]. In particular, hypertension was defined as office systolic blood pressure (BP) ≥ 140 and/or diastolic BP ≥ 90 mmHg or current antihypertensive drug treatment [13]. Diabetes was defined according to the history of diabetes (fasting plasma glucose ≥ 126 mg/dL or hemoglobin A1c ≥ 48 mmol/mol at baseline examination or treatment with antidiabetic drugs) [14]. The estimated glomerular filtration rate (eGFR) was calculated using the standard formula [15]. The overt kidney dysfunction was defined as eGFR equals below 60 mL/min per 1.73 m^2^ [16]. The SUA/sCr ratio (expressed in Units) was calculated according to the formula SUA (mg/dL) divided by serum creatinine (mg/dL). The triglycerides/HDL-cholesterol ratio (expressed in Units) was calculated using the following formula: triglycerides (mg/dL) divided by HDL-cholesterol (mg/dL). CV mortality for incident events was evaluated at the end of the follow-up [12].

### 2.3. Statistical Analysis

The statistical analyses were performed using the SPSS software (version 23, SPSS Inc., Chicago, IL, USA) and the statistical package R (version 4.3.1). Because eGFR, SUA, creatinine, glucose, total cholesterol, triglycerides, and HDL-cholesterol were non-normal distributed, log-transformed values were used in the analyses. Bivariate relationships between the variables under investigation were evaluated by Spearman’s correlation analysis. To analyze the type of association between SUA/sCr (as a continuous variable) and CV mortality, restricted cubic splines (RCS) regression models with 4 knots (5th-reference, 35th, 65th, and 95th percentiles) were utilized. For the present study, the sample was stratified by participants with SUA/sCr ratio above or below 5.35 Units, according to the previously detected threshold for CV mortality [10]. To evaluate differences among groups’ characteristics, the analysis of variance (ANOVA) was used for continuous data, and the chi-squared test was utilized to evaluate differences between categorical variables. To assess the role of baseline SUA/sCr ratio on the risk of CV mortality, Kaplan–Meier survival curves, log-rank tests, and Cox proportional-hazards models were used. The impact of traditional risk factors and that of potential confounding factors of the sample (*p*-value < 0.2, relating to the comparison between those who died and those who did not) was explored by multivariate models adjusted for baseline age, gender, cigarette smoking, body mass index (BMI), hypertension status, total cholesterol, HDL-cholesterol, triglycerides, blood glucose, and statin use. The proportional hazard (PH) assumption was assessed by visual inspection of Kaplan-Meyer curves. Furthermore, separate analyses stratified by baseline kidney function were also carried out after the identification of two groups: one including participants with evidence of overt kidney dysfunction (KD[+], defined as eGFR ≤ 60 mL/min per 1.73 m^2^) and the other one including participants without evidence of overt kidney dysfunction (KD[−], defined as eGFR > 60 mL/min per 1.73 m^2^). Finally, in consideration of the results and the strong interaction among kidney function, SUA and CV mortality rate, we identified an additional cut-off point (Youden’s index) for KD[+] by the receiver-operating characteristic (ROC) analysis and the area under the curve (AUC), with its 95% confidence interval (CI), to assess the ability of SUA/sCr ratio on CV mortality in this subgroup (cut-point: 7.50 Units). The results are reported as mean (or geometric mean) with SD, percentages, or hazard ratio (HR) and 95%CI (Bootstrap CI, 1000 iterations) unless otherwise indicated. Two-sided *p* values < 0.05 were considered statistically significant.

## 3. Results

The baseline characteristics of the 2230 participants included are reported in Table 1.

**Figure 1:**
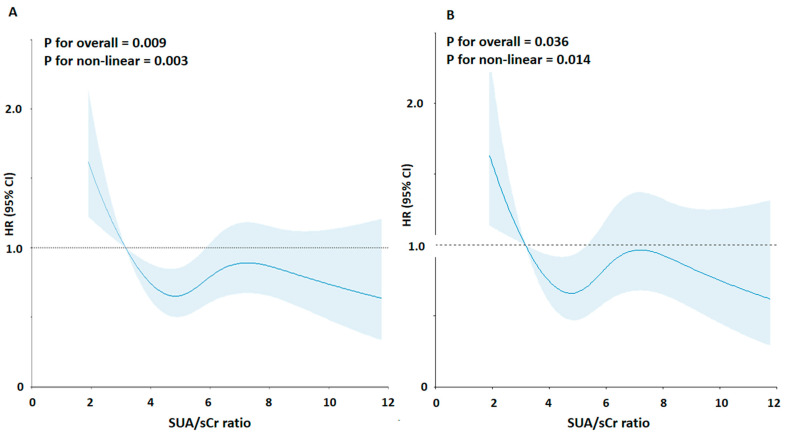
Association between the SUA/sCr ratio and risk of cardiovascular mortality using a Restricted Cubic Spline Regression Model. Solid lines indicate hazard ratios (HRs), and shadow shapes indicate 95% confidence intervals (CIs). (**A**) Unadjusted; (**B**) Adjusted for age, gender, body mass index, cigarette smoking, hypertension status, total cholesterol, triglycerides, blood glucose, and statin use.

The mean age at baseline was 65.0 years; 51.2% were men, 45.7% of the participants were overweight, 32.9% were obese, 18.3% were smokers, and 76.2% were hypertensive (47% on regular antihypertensive treatment). The prevalence of high SUA/sCr ratio was 53.5% according to the 5.35 Units threshold. The analysis of the correlation between the SUA/sCr ratio and the most relevant characteristics of participants at baseline showed a significant association with BMI (r = 0.18), eGFR (r = 0.30), blood glucose (r = −0.06) and triglycerides (r = 0.08), and as expected with SUA (r = 0.70) and creatinine (r = −0.36); by contrast, no association was found with age, systolic and diastolic BP, total cholesterol, HDL-cholesterol, and triglycerides/HDL-cholesterol ratio (*p* > 0.05) . During a median follow-up of 9.2 years (110 months, 25th–75th: 60–144 months), 671 (30.1%) all-cause deaths occurred, 380 of which were due to primary CV causes (acute myocardial infarction = 113, heart failure = 98, stroke = 86, and hypertensive complications = 83). RCS regression model detected a non-linear relationship between SUA/sCr ratio and CV mortality (test for overall: *p* < 0.001, test for non-linearity: *p* < 0.001) (Figure 1A). The shape of the association was also confirmed after adjustment for main confounders (Figure 1B).

The differences in baseline characteristics between participants stratified by the SUA/sCr ratio, defined according to thresholds of 5.35 Units, are also reported in Table 1. Diabetic individuals with SUA/sCrea ratio > 5.35 Units had significantly higher BMI, SUA, triglycerides and eGFR, and higher prevalence of obesity, and hypertension, than those with SUA/sCr ratio below 5.35 Units. In addition, individuals with SUA/sCr ratio above 5.35 Units had significantly lower serum creatinine and prevalence of kidney dysfunction and cigarette smoking.

Participants with SUA/sCr ratio of more than 5.35 had a non-significant higher incidence of CV mortality than participants with an SUA/sCr ratio below 5.35 (18.1% vs. 15.9%, *p* = 0.17). To assess the multivariate-adjusted models, we also evaluated the differences in baseline characteristics between those who died and those not (Table 2).

As expected, those who died had a worse cardio-metabolic profile. The Kaplan-Meier curves showed an overlap of the curves (log-rank test: 1.288, *p* = 0.26) (Supplemental Figure S1). The Cox regression analysis confirmed the non-significant difference in the risk of CV mortality between the two groups (>5.35 vs. ≤5.35 Units: HR: 1.12, 95%CI: 0.91–1.38).

**Figure 2:**
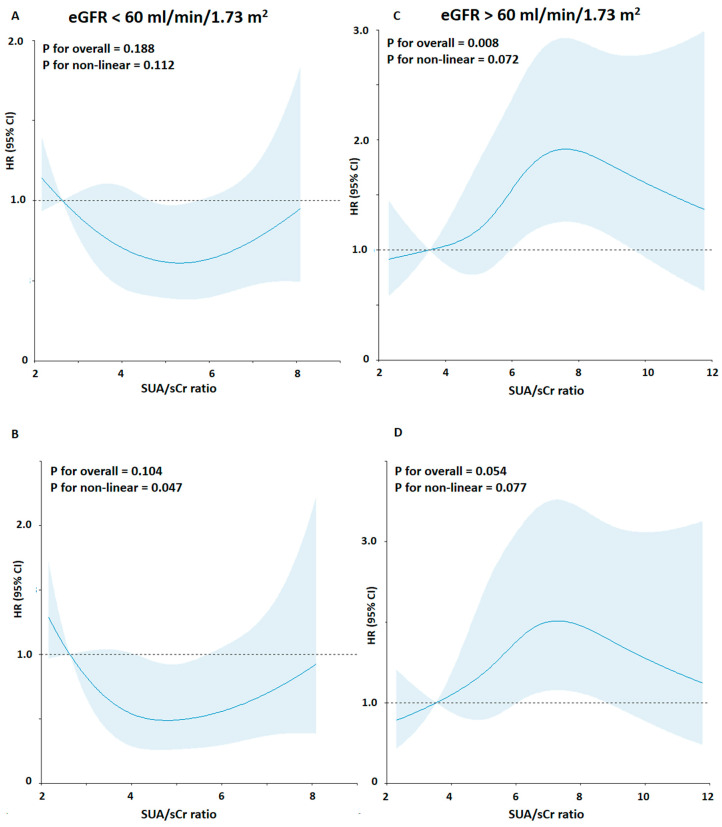
Association between the SUA/sCr ratio and risk of cardiovascular mortality using a Restricted Cubic Spline Regression Model stratified by kidney function. Solid lines indicate hazard ratios (HRs), and shadow shapes indicate 95% confidence intervals (CIs). (**A**,**C**) Unadjusted; (**B**,**D**) Adjusted for age, gender, body mass index, cigarette smoking, hypertension status, total cholesterol, triglycerides, blood glucose, and statin use.

Next, in consideration of these results, the interaction among SUA, kidney function, and CV mortality rate, and the shape of the association between SUA/sCr ratio and CV mortality risk, we explored the relationship between SUA/sCr ratio and CV mortality stratifying by kidney function (Figure 2) (Table 3).

In the KD[−] group (*n* = 1697), participants with SUA/sCrea ratio > 5.35 Units had significantly higher age, BMI, systolic BP, SUA, and eGFR, higher prevalence of hypertension and statin use, and lower serum creatinine and prevalence of smokers than those with SUA/sCr ratio below 5.35 Units.

**Figure 3:**
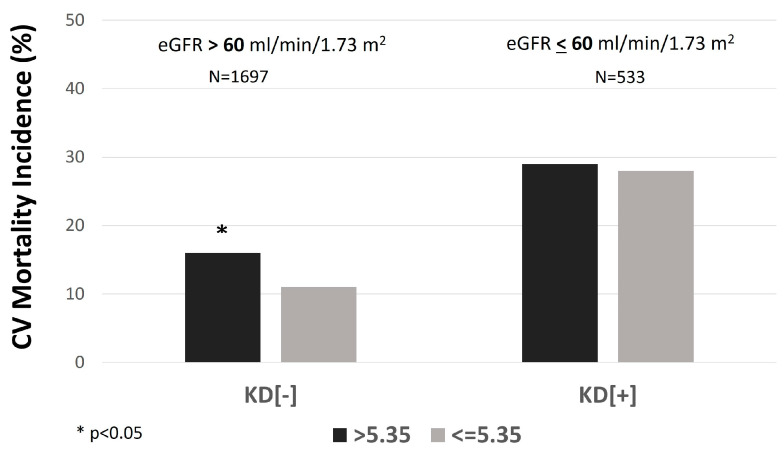
The incidence rate of cardiovascular (CV) mortality in individuals with serum uric acid (SUA)/creatinine (sCr) ratio > 5.35 vs. ≤5.35 mg/dL, stratified by kidney function. KD[+]: eGFR ≤ 60 mL/min per 1.73 m^2^; KD[−]: eGFR > 60 mL/min per 1.73 m^2^.

Moreover, the participants of the KD[−] group with a SUA/sCr ratio of more than 5.35 Units had a significantly higher incidence of CV mortality than participants with a SUA/sCr ratio below 5.35 Units (16.0% vs. 10.4%, *p* = 0.001) (Figure 3).

**Figure 4:**
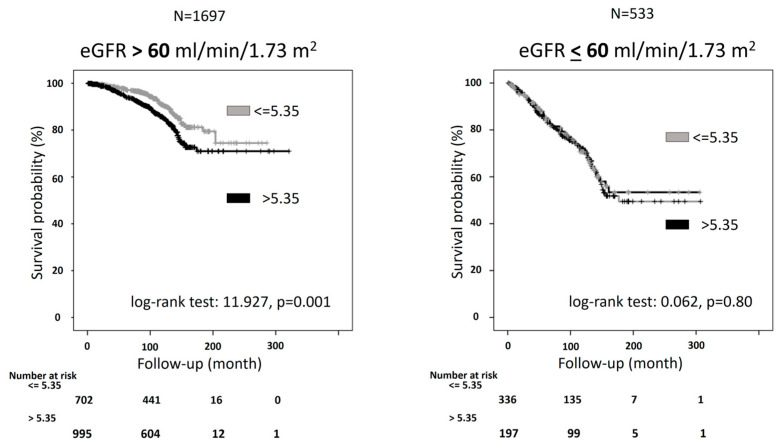
Kaplan-Meier curves for cardiovascular (CV) mortality for people with serum uric acid (SUA)/Creatinine (sCr) ratio lower or above 5.35 Units stratified by kidney function. KD[+]: eGFR ≤ 60 mL/min per 1.73 m^2^; KD[−]: eGFR > 60 mL/min per 1.73 m^2^.

In the KD[+] group (*n* = 533), individuals with a SUA/sCr ratio above 5.35 Units had significantly higher BMI, SUA, total cholesterol, and eGFR and lower serum creatinine than those with a ratio below 5.35 Units. Moreover, the contrary of the other group, in the KD[+] group, there was no significantly higher rate of CV mortality in participants with a SUA/sCr ratio of more than 5.35 Units compared with the other group (28.9% vs. 27.5%, *p* = 0.72) (Figure 3). The Kaplan-Meier curves for CV mortality in KD[−] showed that participants with values above 5.35 Units had a significantly higher probability of CV mortality than those with SUA/sCr ratio below 5.35 Units (log-rank test: 11.927, *p* = 0.001) (Figure 4).

The inspection of Kaplan-Meyer curves did not detect a PH assumption violation. The Cox regression analysis confirmed the predictive role of the CV mortality cut-off, which showed a greater risk of CV mortality in participants with SUA/sCr ratio above with respect to below 5.35 Units (Table 4).

This predictive role was also detected after adjustment for some potential confounders (Table 4). The relationship was also confirmed in a model including insulin resistance, expressed by triglyceride/HDL-cholesterol ratio instead of triglycerides and HDL-cholesterol (HR: 1.58, 95%CI: 1.11–2.25). On the other hand, as expected, the Kaplan-Meier curves for CV mortality in KD[+] showed an overlap of the two curves of the survival (log-rank test: 0.062, *p* = 0.80) (Figure 3). The trend was confirmed by the Cox regression analysis, which indicated no significant risk difference between the two groups (Table 4).

Moreover, also the results from further stratification by different levels of overt kidney dysfunction confirmed this trend in KD[+] (eGFR 45–60 mL/min/1.73 m^2^, *n* = 387, 27.2% vs. 22.8%, *p* = 0.32; eGFR < 45 mL/min/1.73 m^2^, *n* = 146, 41.7% vs. 35.8%, *p* = 0.58).

Given these results and the reasons mentioned above, we assessed a different cut-off point for KD[+] (7.50 Units). In this group, the Kaplan-Meier curves for CV mortality showed that participants with values above 7.50 Units had a significantly higher probability of CV mortality than those with SUA/sCr ratio below 7.50 Units (log-rank test: 4.165, *p* = 0.04) (Supplemental Figure S2). The inspection of Kaplan-Meyer curves did not detect a PH assumption violation. The Cox regression analysis confirmed the predictive role of this CV mortality cut-off value, which showed a greater risk of CV mortality in participants with SUA/sCr ratio above with respect to below 7.50 Units (Table 5).

This predictive role was also detected after adjustment for some potential confounders (Table 5). The association was also confirmed in a model including insulin resistance, expressed by triglyceride/HDL-cholesterol ratio instead of triglycerides and HDL-cholesterol (HR: 2.83, 95%CI: 1.41–5.69).

## 4. Discussion

The main findings of this study showed a non-linear association between baseline SUA/sCr ratio and risk of CV mortality in diabetic participants. Moreover, the SUA/sCr ratio threshold previously proposed by the URRAH study group [10] was not predictive of CV mortality in the whole diabetic population. In consideration of this result and the strong relevance of kidney function on SUA and CV mortality rate, we analyzed the sample stratified into two groups by the presence or absence of kidney dysfunction. The SUA/sCr ratio of 5.35 Units is predictive for CV mortality in a diabetic population with normal kidney function (eGFR > 60 mL/min per 1.73 m^2^), revealing that values of SUA/sCr ratio higher than 5.35 were associated with a 39% increased risk of CV mortality in this group, independently of potential confounders, among which lipid and glucose profile, and insulin resistance. By contrast, this cut-point was not predictive of CV mortality in diabetic participants with kidney dysfunction (<60 mL/min per 1.73 m^2^). However, in this group, a newly detected cut-point (7.50 Units) was associated with the rate of CV mortality and values of the SUA/sCr ratio above 7.50 were associated with a 79% increased risk, independently of hypertension, lipid, and glucose profile, and insulin resistance. These results are strengthened by the large study population being highly representative of the general population with diabetes, the long follow-up period, and no bias due to the pharmacological treatment of hyperuricemia.

These results are in agreement with our previous data on diabetic participants of the URRAH study, which showed a direct relationship between SUA and CV mortality risk [14]. In addition, our results are in line with data on the relationship between the SUA/sCr ratio and CV risk in diabetic populations. Indeed, SUA/sCr was an independent risk factor for the progression of diabetic nephropathy in a large diabetic Chinese population [17]. Similar results were found in a study involving a sample of rural elderly diabetic patients, in which the SUA/sCr ratio had a predictive role in the decline in kidney function during a follow-up of 6 months [18]; likewise, SUA/sCr ratio was also an independent risk factor for kidney disease in diabetic patients with normo-albuminuria [19]. On the other hand, although the values above 5.35 were associated with a slightly higher rate of CV mortality in diabetic participants with kidney dysfunction, these values were not predictive of CV mortality in this group. This inconsistency in the predictive role of SUA/sCr ratio between KD[−] and KD[+] may be due to the strong influence of kidney function on the index and the rate of CV mortality, thus to the subsequent substantial inverse non-linear relationship between the SUA/sCr ratio and CV mortality risk in this sample, to the small sample of participants with kidney dysfunction and to the difference in the index values (as continuous item) between KD[+] and KD[−]. Indeed, in the whole sample, the SUA/sCr ratio was significantly higher in KD[−] than in KD[+], as expected. Noteworthy, after stratification by kidney dysfunction, the SUA/sCr ratio was significantly higher in those who died than not in KD[−], whereas the index distribution was not different in KD[+]. Furthermore, these data are in agreement with those of a recent study on a general population that found an elevated risk effect of hyperuricemia associated with CV mortality in individuals with normal kidney function rather than with reduced kidney function [20]. Likely, in normal kidney function, hyperuricemia due to the overproduction of uric acid is a more harmful CV risk factor than hyperuricemia resulting from reduced kidney excretion of SUA; the interaction between diabetes and kidney dysfunction would explain a high mortality rate in participants with kidney dysfunction. Hence, in KD[+], the direct effect of SUA on CV mortality was affected by kidney dysfunction. Moreover, the biological mechanism(s) linking SUA with CV disease have been hypothesized and include oxidative stress, inflammation, and arterial stiffness [21]. SUA is also an indicator of kidney function: SUA levels are influenced by kidney clearance function, and higher SUA levels are observed in patients with poor kidney function. Therefore, the SUA/sCr ratio, since it reflects SUA levels normalized to kidney function, could serve as a more accurate indicator of CV disease.

In this context, our results in the diabetic sample with preserved kidney function confirmed the predictive role of the ratio threshold found in the whole URRAH sample [10], highlighting the role of kidney-independent metabolic overproduction of SUA on CV risk. The apparent discrepancies with participants with kidney dysfunction may be justified by the substantial superiority of the kidney function on CV mortality rate in this subgroup of diabetic participants and the negligible prevalence of kidney dysfunction in the large sample of the general population participating in the URRAH study [10]. Furthermore, despite the cardio-metabolic risk profile being worse in those with a ratio above 5.35 Units, the potential independent predictive role of the SUA/sCr ratio on CV mortality may not be disentangled in this sample at high CV risk also for the strong interaction among diabetes, kidney dysfunction, and SUA [11,17,19].

Nevertheless, our study has some limitations: (i) the URRAH study enrolled only white participants. Thus, its results are not generalizable to other ethnicities; (ii) the study design is observational. Hence, it did not allow the establishment of the cause-relationship; (iii) the analysis was based on a single SUA and sCr measurement; however, these parameters have little pre-analytical biological variability.

## 5. Conclusions

The results of the present study show for the first time that the SUA/sCr ratio is predictive of CV mortality in people with diabetes. In particular, because the kidney function strongly affects the values of SUA/sCr ratio and the rate of CV mortality, the main findings of the study indicate two different thresholds: 5.35 Units (previously proposed by the URRAH study group [10]) for diabetic participants with preserved kidney function, and 7.50 Units for diabetic participants with overt kidney dysfunction. Although this population is already at high risk for the presence of diabetes, these cut-offs allow us to identify those at greater CV risk in addition to the risk due to diabetes. These data highlight the different predictive roles of SUA (and its interaction with kidney function) in the CV risk, pointing out the difference in metabolic- and kidney-dependent SUA levels also in diabetic individuals, hence the importance of the stratification by kidney function. Given the strong interaction between SUA and kidney function, this index is more complete than SUA alone in predicting CV mortality. For these reasons, these results make the SUA/sCr ratio a low-cost, reliable marker applicable to routine clinical practice of diabetes care. Nonetheless, further studies are needed to support our conclusions.

## Figures and Tables

**Table 1:** Baseline characteristics of all the study participants and stratified according to the SUA/sCr ratio threshold predictive for cardiovascular mortality.

Variables	Total Sample	SUA/sCr Ratio	
		≤5.35	>5.35
No. of participants	2230	1038	1192
Age (years)	65.0 (12.7)	64.6 (12.9)	65.3 (12.6)
Gender (M/F%)	51.2/48.8	52.3/47.7	50.2/49.8
Cigarette Smoking (%)	18.3	20.0	16.8 *
BMI (kg/m^2^)	28.5 (4.6)	27.8 (4.5)	29.1 (4.6) *
Normal-weight (%)	21.4	26.4	17.0
Overweight (%)	45.7	45.5	46.0
Obesity (%)	32.9	28.1	37.0 *
Systolic BP (mmHg)	151.5 (26.1)	150.6 (26.0)	152.3 (26.1)
Diastolic BP (mmHg)	85.3 (12.5)	85.1 (12.4)	85.5 (12.6)
Hypertension (%)	76.2	74.3	77.9 *
Creatinine (mg/dL) ^a^	0.93 (1.3)	1.01 (1.3)	0.88 (1.2) *
eGFR (mL/min/1.73 m^2^) ^a^	72.4 (1.4)	66.1 (1.4)	77.6 (1.3) *
Kidney dysfunction-eGFR ≤ 60 mL/min/1.73 m^2^ (%)	23.9	32.4	16.5 *
Serum Uric Acid (mg/dL) ^a^	5.01 (1.3)	4.2 (1.3)	5.9 (1.2) *
Glucose (mg/dL) ^a,b^	134.9 (1.4)	138.0 (1.4)	131.8 (1.3)
Total cholesterol (mg/dL) ^a,c^	208.9 (1.2)	204.2 (1.2)	208.9 (1.2)
Triglycerides (mg/dL) ^a,c^	134.9 (1.7)	131.8 (1.7)	141.2 (1.7) *
HDL-cholesterol (mg/dL) ^a,d^	46.8 (1.3)	46.8 (1.3)	46.9 (1.2)
Triglycerides (mg/dL)/HDL-cholesterol (mg/dL) ^d^	3. 7 (3.1)	3.5 (2.9)	3.8 (3.3)
Statin use (%)	6.7	5.6	7.7

Data are expressed as means (SD); BMI: body mass index; BP: Blood Pressure; eGFR: estimated glomerular filtration rate. Overweight was defined as a BMI between 25 and 29.9 kg/m^2^ and obesity as BMI ≥ 30 kg/m^2^. Hypertension was defined as office systolic BP ≥ 140 and/or diastolic BP ≥ 90 mmHg or current antihypertensive drug treatment. ^a^ Data are expressed as geometric mean (SD); ^b^ sample reduced by 15%; ^c^ sample reduced by 5%; ^d^ sample reduced by 20%; * >5.35 vs. ≤5.35: *p* < 0.05.

**Table 2:** Baseline characteristics stratified by cardiovascular mortality.

Variables	Cardiovascular Mortality	
	No	Yes
No. of participants	1850	380
Age (years)	63.3 (12.6)	73.2 (9.5) *
Gender (M/F%)	53/47	41/59 *
Cigarette Smoking (%)	20	11 *
BMI (kg/m^2^)	28.6 (4.6)	28.3 (4.7)
Hypertension (%)	74	86 *
Glucose (mg/dL) ^a,b^	131.8 (1.3)	147.9 (1.4) *
Total cholesterol (mg/dL) ^a,c^	204.2 (1.2)	208.9 (1.2)
Triglycerides (mg/dL) ^a,c^	134.9 (1.7)	134.9 (1.6)
HDL-cholesterol (mg/dL) ^a,d^	46.8 (1.3)	48.9 (1.3)
Triglycerides (mg/dL)/HDL-cholesterol (mg/dL) ^d^	3.7 (3.2)	3.6 (3.0)
Statin use (%)	8	2 *

Data are expressed as means (SD); BMI: body mass index; Hypertension was defined as office systolic BP ≥ 140 and/or diastolic BP ≥ 90 mmHg or current antihypertensive drug treatment. ^a^ Data are expressed as geometric mean (SD); ^b^ sample reduced by 15%; ^c^ sample reduced by 5%; ^d^ sample reduced by 20%. * yes vs. no: *p* < 0.05.

**Table 3:** Baseline characteristics of the study participants stratified by kidney dysfunction (KD) and according to the SUA/sCr ratio threshold, which is predictive for cardiovascular mortality.

Variables	KD [−] > 60 mL/min/1.73 m^2^		KD [+] ≤ 60 mL/min/1.73 m^2^	
	SUA/sCr Ratio		SUA/sCr Ratio	
	≤5.35	>5.35	≤5.35	>5.35
No. of participants	702	995	336	197
Age (years)	60.9 (12.4)	63.9 (12.6) *	72.4 (10.0)	72.6 (9.8)
Gender (M/F%)	59.9/40.1	55.4/44.6	36.6/63.4	23.9/76.1 *
Cigarette Smoking (%)	23.1	17.6 *	13.7	12.7
BMI (kg/m^2^)	27.8 (4.4)	29.1 (4.6) *	27.9 (4.7)	29.3 (4.5) *
Normal-weight (%)	25.0	17.5	29.0	14.4
Overweight (%)	46.9	46.0	42.6	45.6
Obesity (%)	28.1	36.5 *	28.4	40.0 *
Systolic BP (mmHg)	149.7 (26.1)	151.4 (26.2) *	154.6 (25.5)	156.6 (25.0)
Diastolic BP (mmHg)	84.9 (12.1)	85.4 (12.2)	85.4 (12.9)	86.1 (14.4)
Hypertension (%)	69.5	76.2 *	84.2	86.3
Creatinine (mg/dL) ^a^	0.91 (1.2)	0.84 (1.2) *	1.26 (1.3)	1.11 (1.1) *
eGFR (mL/min/1.73 m^2^) ^a^	79.4 (1.2)	85.1 (1.3) *	44.7 (1.3)	51.3 (1.1) *
Serum Uric Acid (mg/dL) ^a^	3.9 (1.3)	5.8 (1.2) *	4.8 (1.3)	7.1 (1.2) *
Glucose (mg/dL) ^a,b^	135.8 (1.4)	131.0 (1.3)	141.2 (1.4)	138.6 (1.3)
Total cholesterol (mg/dL) ^a,c^	208.9 (1.2)	208.4 (1.2)	200.0 (1.24)	210.2 (1.2) *
Triglycerides (mg/dL) ^a^	125.9 (1.7)	134.9 (1.7) *	141.2 (1.7)	162.2 (1.7) *
HDL-cholesterol (mg/dL) ^a,d^	46.8 (1.3)	47.9 (1.3)	46.8 (1.4)	46.8 (1.3)
Triglycerides(mg/dL)/HDL-cholesterol (mg/dL) ^d^	3.7 (2.8)	3.6 (3.3)	3.9 (3.1)	4.4 (3.3)
Statin use (%)	4.0	7.6 *	8.6	8.2

Data are expressed as means (SD): BMI: body mass index; BP: Blood Pressure; eGFR: estimated glomerular filtration rate. Overweight was defined as a BMI between 25 and 29.9 kg/m^2^ and obesity as BMI ≥ 30 kg/m^2^. Hypertension was defined as office systolic BP ≥ 140 and/or diastolic BP ≥ 90 mmHg or current antihypertensive drug treatment. ^a^ Data are expressed as geometric mean (SD); ^b^ sample reduced by 15%; ^c^ sample reduced by 5%; ^d^ sample reduced by 20%; * >5.35 vs. ≤5.35: *p* < 0.05.

**Table 4:** Cox-regression analysis of the risk of cardiovascular mortality according to kidney dysfunction (KD).

	KD[−]	KD[+]
	SUA/sCr Ratio >5.35 vs. ≤5.35	SUA/sCr Ratio >5.35 vs. ≤5.35
	HR (95% CI *)	HR (95% CI *)
Unadjusted	1.62 (1.23–2.20) ^§^	0.96 (0.66–1.35)
Multivariable Model 1 ^a^	1.32 (1.01–1.74) ^§^	1.01 (0.72–1.42)
Multivariable Model 2 ^b^	1.35 (1.01–1.81) ^§^	0.98 (0.69–1.40)
Multivariable Model 3 ^c^	1.61 (1.15–2.26) ^§^	0.98 (0.65–1.48)
Multivariable Model 4 ^d^	1.58 (1.11–2.23) ^§^	0.97 (0.64–1.49)

Hypertension was defined as office systolic BP ≥ 140 and/or diastolic BP ≥ 90 mmHg or current antihypertensive drug treatment; HR: Hazard Ratio; KD[−]: >60 mL/min/1.73 m^2^; KD[+]: ≤60 mL/min/1.73 m^2^; SUA/sCr ratio: serum uric acid/serum creatinine ratio; * Bootstrap confidence intervals (1000 iterations); ^§^ *p* < 0.05. ^a^ Model 1 adjusted for baseline age, gender, body weight, hypertension, and cigarette smoking. ^b^ Model 2 adjusted for Model 1 plus baseline (log)total cholesterol, (log)triglycerides, and statin use (sample reduced by 5%). ^c^ Model 3 adjusted for Model 2 plus baseline (log) blood glucose (sample reduced by 15%). ^d^ Model 4 adjusted for Model 3 plus baseline (log)HDL-cholesterol (sample reduced by 20%).

**Table 5:** Cox-regression analysis of the risk of cardiovascular mortality patients with kidney dysfunction (KD).

	KD[+] <60 mL/min/1.73 m^2^
	SUA/sCr ratio ≥7.50 vs. <7.50
	HR (95% CI *)
Unadjusted	1.79 (1.02–3.17) ^§^
Multivariable Model 1 ^a^	1.92 (1.06–3.45) ^§^
Multivariable Model 2 ^b^	2.92 (1.46–5.86) ^§^
Multivariable Model 3 ^c^	2.90 (1.44–5.86) ^§^

Hypertension was defined as office systolic BP ≥ 140 and/or diastolic BP ≥ 90 mmHg or current antihypertensive drug treatment; HR: Hazard Ratio; KD[+]: ≤60 mL/min/1.73 m^2^; SUA/sCr ratio: serum uric acid/serum creatinine ratio; * Bootstrap confidence intervals (1000 iterations); ^§^ *p* < 0.05. ^a^ Model 1 adjusted for baseline age, gender, body weight, hypertension, and cigarette smoking. ^b^ Model 2 adjusted for Model 1 plus baseline (log)total cholesterol, (log)triglycerides, statin use, (log) blood glucose (sample reduced by 30%). ^c^ Model 3 adjusted for Model 2 plus baseline (log)HDL-cholesterol (sample reduced by 20%).

## Data Availability

The data presented in this study are available on a specific request from the corresponding author. The data are not publicly available.

## Supplementary Materials

The following supporting information can be downloaded at: https://www.mdpi.com/article/10.3390/metabo14030164/s1, Supplemental Methods: Additional information on methods; Figure S1: Kaplan-Meier curves for cardiovascular (CV) mortality for people with serum uric acid (SUA)/Creatinine (sCr) ratio lower or above 5.35 Units; Figure S2: Kaplan-Meier curves for cardiovascular (CV) mortality for people with kidney dysfunction and stratified by serum uric acid (SUA)/Creatinine (sCr) ratio lower or above 7.50 Units.
