# Supplementary material for: Serum Uric Acid/Serum Creatinine Ratio and Cardiovascular Mortality in Diabetic Individuals—The Uric Acid Right for Heart Health (URRAH) Project
Source: Metabolites. 2024 Mar 14;14(3):164. doi: 10.3390/metabo14030164 (PMC10972048; doi:10.3390/metabo14030164)
Supplement: Supplementary file 1 [file metabolites-14-00164-s001.zip › metabolites-2880496-supplementary.pdf]

Supplemental Methods

Study Population

The URRAH database is a multicenter retrospective, observational cohort study, which involves data from several cohorts distributed in almost all the Italian regions (age: 18-95 years). The URRAH study was performed according to the Declaration of Helsinki for Human Research (41st World Medical Assembly, 1990). Approval was sought from the Ethics Committee of the coordinating center at the Division of Internal Medicine of the University of Bologna (no. 77/2018/Oss/AOUBo). Informed consent was obtained from all individuals upon recruitment. From an updated URRAH database (a total of 3,157 diabetic participants at baseline - 12% of the whole population), 2,230 diabetic participants were considered for the purpose of the present study, after sequential exclusion of participants without a complete database (n=903) and those on hypouricemic therapy at baseline (n=24).

Examination Procedures

A nationwide Italian database was constructed by collecting individual data on patients with anthropometric, biochemical measurements, blood pressure (BP) and heart rate assessment, and clinical history information. Hypertension was defined as office systolic BP ≥ 140 and/or diastolic BP ≥90 mmHg or current antihypertensive drug treatment. Diabetes was defined according to history of diabetes (fasting plasma glucose ≥126 mg/ dL, or hemoglobin A1c ≥ 48 mmol/mol at baseline examination or treatment with antidiabetic drugs). Body mass index (BMI) was calculated according to the formula weight (kg)/height2 (m). Overweight was defined as BMI between 25 and 29.9 kg/m2, and obesity as BMI ≥ 30 kg/m2. The estimated glomerular filtration rate (eGFR) was calculated by standard formula. Renal dysfunction was defined as eGFR equal-below 60 ml/min per 1.73 m^2. Serum uric acid (SUA) / serum creatinine (sCr) ratio (expressed in Units) was calculated according to the formula SUA (mg/dl) divided by sCr (mg/dl).

Outcomes Assessment

Cardiovascular mortality for incident events due to acute myocardial infarction, heart failure, stroke, and hypertensive complications was evaluated at the end of the follow-up. Information on patients who had died was obtained from hospital records or death certificates. Mortality from CV disease was coded according to the International Classification of Diseases, Tenth Revision – ICD10.

Figure S1

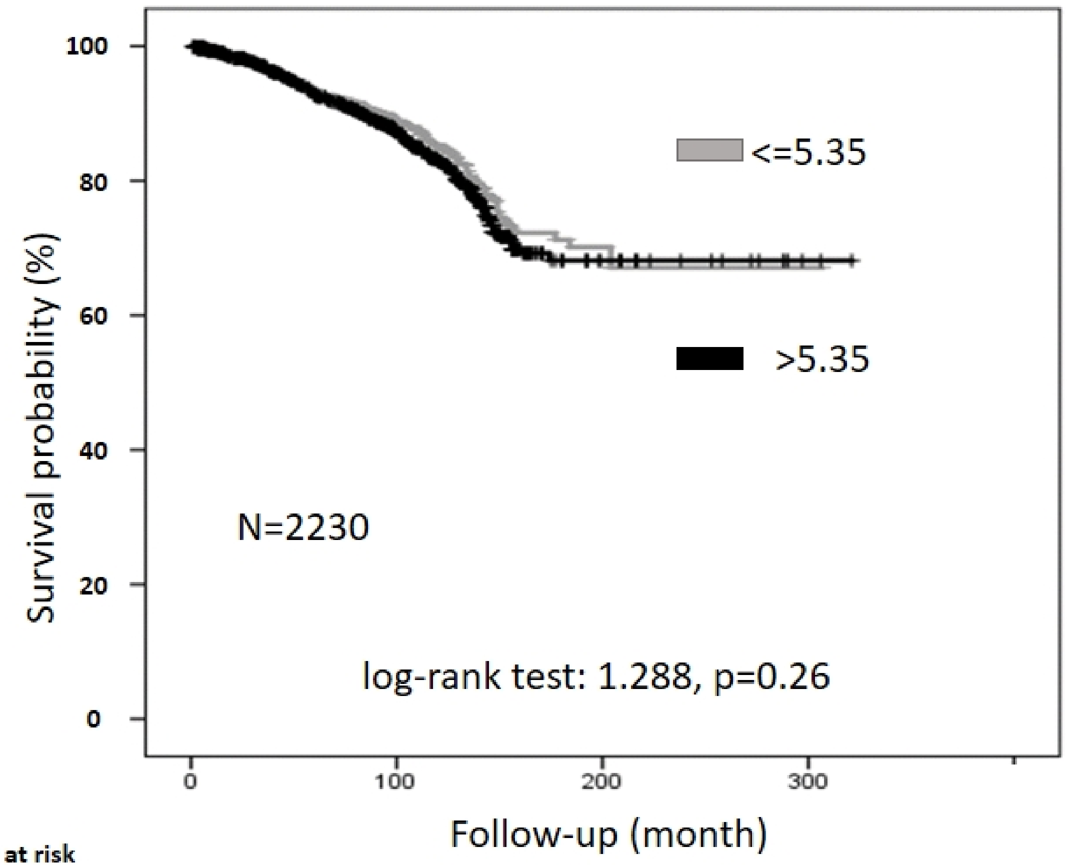

Number at risk

**<= 5.35**				
	**1038**	**578**	**24**	**2**
**> 5.35**				
	**1192**	**704**	**17**	**2**

Figure S2

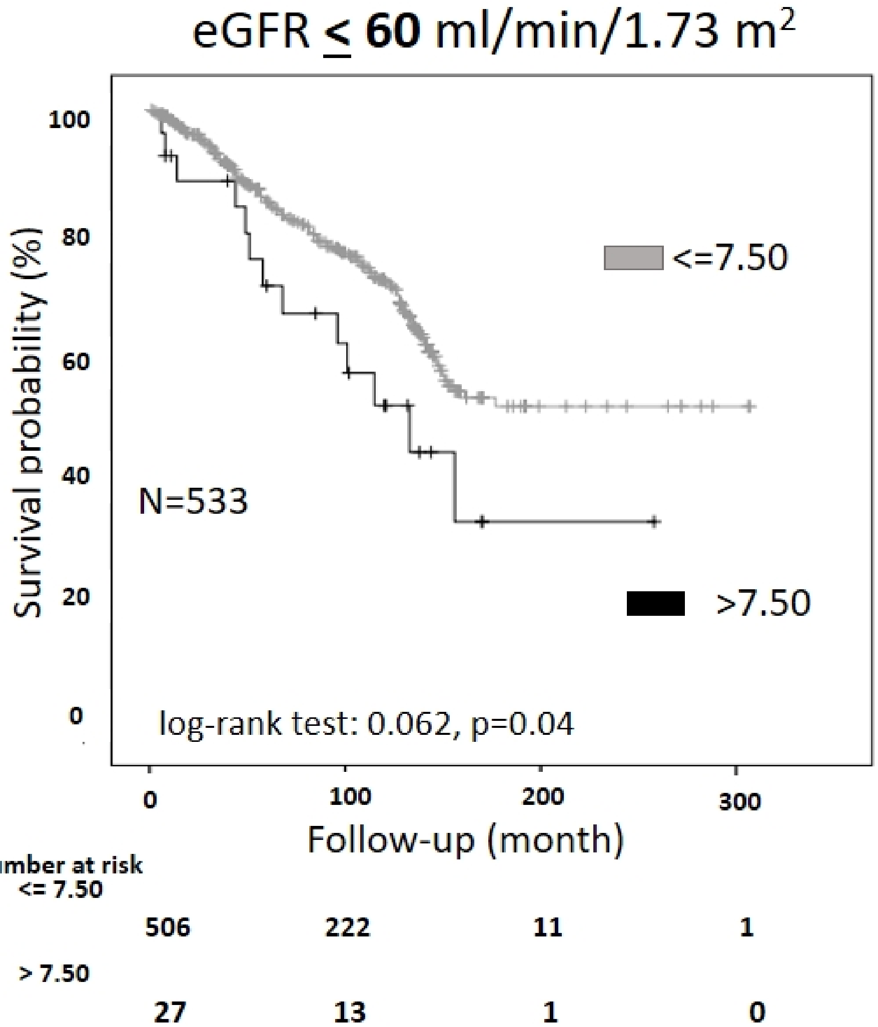

Number at risk

<= 7.50	506	222	11	1
> 7.50	27	13	1	0
